# Social Perception of Autonomous Mobility: A Survey on Public Transport Pilots in Switzerland

**DOI:** 10.1038/s41597-026-06672-y

**Published:** 2026-01-31

**Authors:** Michael Wicki, Himanshu Verma, Julien Reichenbach, Benjamin Nanchen, Jakub Mlynář, Florian Evéquoz, Thomas Bernauer

**Affiliations:** 1 ETH Zürich, Zürich, Switzerland; 2 TU Delft, Delft, Netherlands; 3 HES-SO Valais-Wallis, Sierre, Switzerland

**Keywords:** Interdisciplinary studies, Decision making, Databases, Politics

## Abstract

This data descriptor presents survey data on the social perception of autonomous vehicles (AVs) collected during pilot trials in two Swiss cantons, Schaffhausen and Valais-Wallis. The surveys aimed to capture public acceptance and concerns regarding AVs, focusing on changes in perception before and after the trials. Data was gathered from representative samples in three municipalities per canton, using longitudinal panel surveys in Schaffhausen and a cross-sectional survey in Valais. Possible uses include analyses of public awareness and acceptance of AVs post-trial and investigating regional differences in perceived accessibility and benefits. This dataset offers valuable insights for researchers interested in the integration of AVs into public transport systems. The data enables further analysis and comparison with other regions and contexts.

## Background & Summary

“Autonomous vehicles” (AVs) have been hailed as a technology that has the potential to introduce profound changes in transportation and the organization of urban life more widely^1,2^. Despite extensive efforts, incorporating AVs into the everyday lives of people and into the activity patterns of current societies remains controversial^3–5^. One of the approaches to exploring the social perception of AVs in public transport is research into the public opinion in locations where such a technology was experimentally tested, reflecting how “AI-related technologies move from experimental ‘sandboxes’ and ‘playgrounds’ to routine activities embedded in the structures of everyday life”^6^.

One aspect of the social perception of autonomous technological agents is their social acceptance and public awareness^7–9^. Social acceptance of disruptive technologies, and support for related policies, usually tends to be lower than for other, more incremental innovations^10–12^. This reluctance to accept disruptive technologies is attributed to individuals’ high initial skepticism^13^. Trials can help increase social acceptance and foster legitimacy as implementation of such technology, and in this case AVs, can overcome initial aversion and mitigate biases^14^. Indeed, existing research shows higher acceptance to be expected after the implementation of trials as anxieties are shown to be arbitrary, and individuals grow familiar with the new technology and its regulation^15^. In other words, individuals are likely to modify their evaluation of the technology in question as soon as they are better informed and more familiar with the technology^16^.

AVs have been piloted in numerous street trials across the world. Between 2016 and 2022, according to the Swiss Federal Roads Office (ASTRA), ten pilot trials of AVs for public transportation took place in Switzerland, involving five major public transport operators^17^. This article describes data collected in surveys following pilot testing of a driverless public transport service in two Swiss cantons: Schaffhausen and Valais (Wallis). The surveys presented in this paper examine factors that could explain support for policies aimed at transitions to disruptive technologies, where concerns towards – and especially risk perception of – disruptive technologies have been found to be a precursor in social acceptance-formation processes. It aims to unpack how social acceptance of and concerns towards AVs changed before and after the implementation of two self-driving bus services in Switzerland (SAE level 3; cf.^18^). To assess these questions, we used panel surveys, conducted from 2018 onwards, with a representative sample of residents from three Swiss municipalities for each of the two study cases.

This data descriptor provides background context and relevant usage information about the available data from the panel surveys. It is organized as follows: the remainder of this first section provides information on the two pilot trials and compares them with each other, to provide background for the understanding of the survey design and data collected that are further detailed in sections 2 and 3.

### Pilot trial study in Valais (Wallis)

**Fig. 1:**
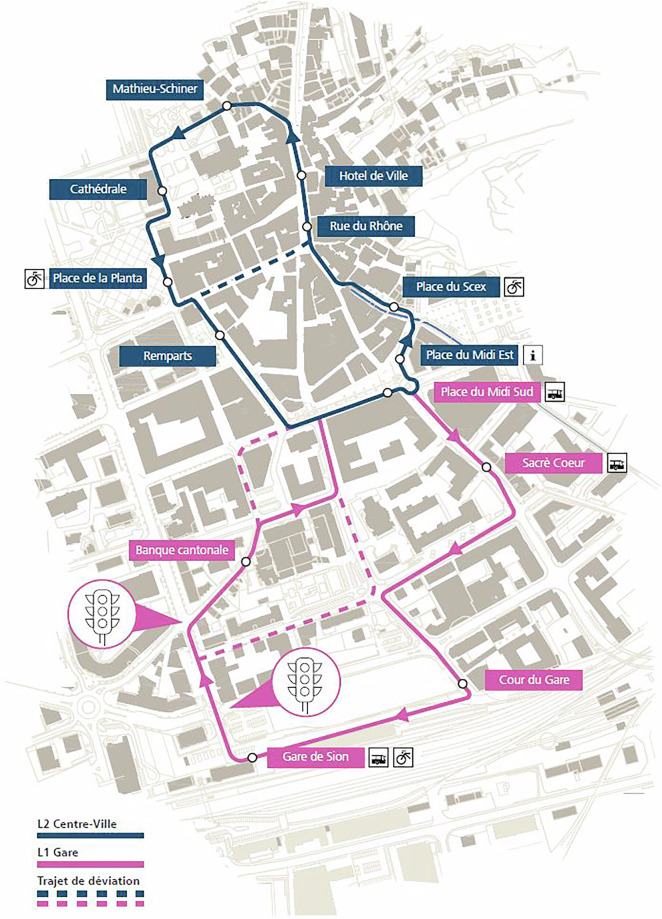
The route in Sion, Valais. “L2” corresponds to the route in the first phase of the trial, “L1” shows its extension in the second phase of the trial.

The pilot of “Smart Shuttle” in the canton of Valais (Wallis) was the first street trial of AVs in Swiss public transport and one of the first worldwide. Conducted in collaboration with the public transport operator PostAuto, its aim was to investigate the technical and practical feasibility of the service, as well as to explore the public perception of this novel technology, and the social conditions and consequences of its use^19–22^. The trial, organized in three subsequent phases, started in June 2016 and finished in October 2021. The location of the first trials in 2016–2017 was in the old town of Sion, which is characterized by mixed traffic with high numbers of pedestrians and by narrow streets (see Fig. 1). In 2018, the route of the shuttle had been extended to the train station in the south. During the testing period, the service was free of charge. The vehicle’s maximum speed was 20 km/h in order to ensure safe operation in the complex urban environment. Only two minor incidents happened during the testing period: collisions with cars in October 2016 and September 2018^23,24^. After concluding the trials in the city center of Sion in 2021, the service was running on demand in the neighboring municipality of Uvrier in 2021–22. There was always a safety attendant present on board, solving emergent troubles on the spot, and guiding passengers or other members of the public in their interactions with the shuttles.

### Pilot trial study in Schaffhausen

**Fig. 2:**
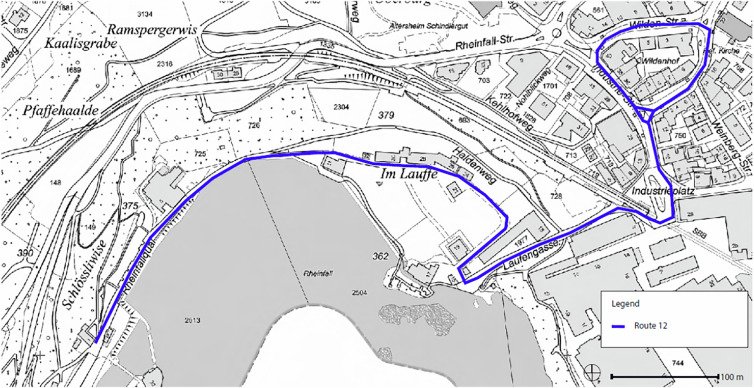
The route in Neuhausen am Rheinfall, Schaffhausen.

The pilot trial study of “Route 12” in the canton of Schaffhausen, specifically in Neuhausen am Rheinfall, represented a pioneering initiative in exploring the perception and acceptance of AVs in public transport services within a Swiss urban context. Specifically, the “Route 12” trial in Neuhausen am Rheinfall aimed to explore the technical viability and practical applicability of a self-driving shuttle service, alongside assessing public opinion and the implications of introducing such a new technology into the community’s daily life by measuring acceptance levels, intention to use, and examining the perceived social impacts arising from their integration into existing public transport networks^25–29^. Launched in March 2018 and concluded in December 2019, the trial served as a significant period for testing and evaluating the integration of autonomous technology into established public transport frameworks. Initially, the AVs were deployed in urban settings, yielding critical insights into the technological and societal implications of such an integration. In June 2019, the route was extended to encompass the Rheinfall waterfalls, transitioning the service into a semi-natural environment and thereby expanding its operational scope and the complexity of its navigational challenges (see Fig. 2). Throughout the duration of the trial, the AV adhered to a maximum speed of 25 km/h, ensuring safety while navigating both urban streets and semi-natural landscapes. An onboard safety attendant was present at all times.

Despite the innovative nature of the service and the challenges associated with autonomous navigation, the trial experienced only one minor incident – a collision with a cyclist in June 2019 –, underscoring the potential for such technology to be safely integrated into public transport systems. This was a particularly noteworthy achievement, considering the shuttle’s journey through areas characterized by mixed traffic and varying environmental conditions. Moreover, the trial’s methodical approach to gathering and analyzing data on social acceptance and operational efficiency provided a robust framework for understanding the broader implications of AVs in public transport.

### Comparison of the two contexts

**Fig. 3:**
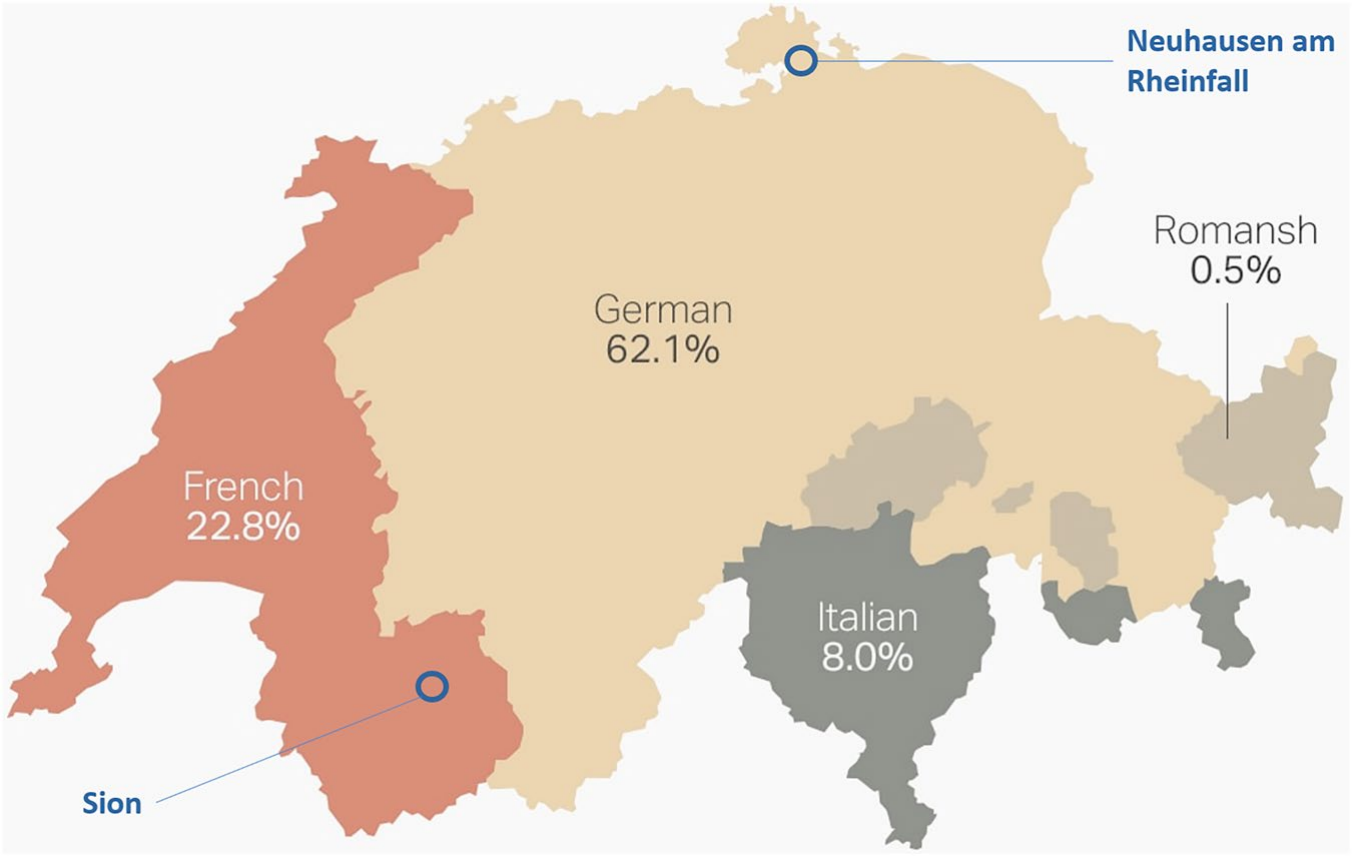
Population partition in terms of languages spoken in Switzerland, and the location of the two municipalities where the pilot trials took place.

**Fig. 4:**
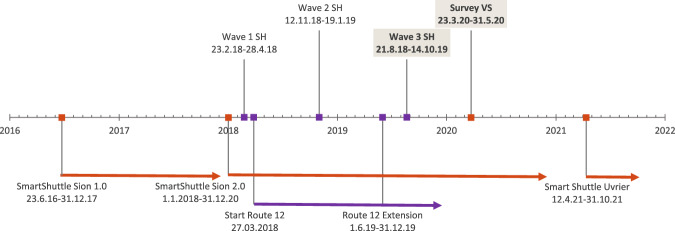
Timeline of the pilot trials and surveys in Schaffhausen (purple) and Valais (orange). The dataset contains data from “Wave 3 SH” and “Survey VS”.

Sion is the capital city of the canton Valais/Wallis with a population of approximately 35 thousand inhabitants and an area of 35 km^2^. It is predominantly French speaking. Neuhausen am Rheinfall (Schaffhausen), on the other hand, is a smaller German-speaking town with a population of approximately 10 thousand inhabitants and area of 8 km^2^. The location of the two municipalities is shown in Fig. 3. The setting and the character of local traffic in both trials of driverless shuttles has also differed: while the pilot in Valais was conducted in a complex urban environment, the trial study in Schaffhausen has commenced in urban environment but was later extended to a semi-natural environment. Both towns are considered to be popular tourist destinations, and many passengers in the pilot trials were therefore not local inhabitants but visitors. The pilot study in Schaffhausen started two years later than the one in Valais, and the duration of the former trial was shorter than the latter one (see Fig. 4). While the AVs in Schaffhausen were tested for 20 months in total, the technology had already been in operation for more than two years in Valais at the time of surveys. Both services were free of charge, and a safety attendant was on board the vehicles in both cantons. The maximum speed of the vehicle in the Schaffhausen pilot was slightly higher than in Valais (25 km/h and 20 km/h, respectively). In both cases, minor incidents happened that have received certain media coverage (two times in Valais and once in Schaffhausen). It is unclear, however, how these incidents could have impacted the public perception of AVs, as neither of the questionnaires included questions related to this particular topic. Both trials provided valuable insights into the technical and practical feasibility of AVs, as well as public perception and social impacts.

## Methods

The aim of the surveys was to understand the development of opinions on the ease of use and effectiveness of AVs as an emerging alternative to public transport. Both studies aimed to assess social acceptance regarding the trial of self-driving shuttle services in the canton of Schaffhausen, with a specific focus on the pilot trial in Neuhausen am Rheinfall, and the canton of Valais, focusing on the pilot trial in Sion. To achieve this, a longitudinal panel survey design was implemented in Schaffhausen, comprising three survey waves spanning from before the initiation of the service through to its operational phase. In parallel with the third wave in Schaffhausen, an identical survey was also conducted in Valais. In each canton, three cities were targeted: the one where the shuttle was running and two other ones in close proximity. We first provide detailed information about the organization of the survey in Schaffhausen, and then additional specifications about the survey in Valais.

### The first and second wave of the Schaffhausen survey

The data from the first and second survey wave in the canton of Schaffhausen^25,26^ is not included in the data set, but we nevertheless provide a description of these studies as an important part of the context in which the comparative study (see 2.2) has been conducted. The initial recruitment for the survey targeted a broad demographic in Schaffhausen, focusing on three municipalities: Neuhausen am Rheinfall, Stein am Rhein, and Thayngen. A total of 8,000 residents were randomly selected from the resident registries, ensuring a representative sample that included a diverse range of age groups, genders, and residency statuses. The participation rate was 17.6% with 1408 responses. The selection criteria were designed to capture a comprehensive view of social acceptance, ranging from those directly affected by the AVs in Neuhausen am Rheinfall to those in neighboring municipalities who might experience indirect impacts or hold general opinions about AVs^25–27^.

The survey was conducted in three waves to monitor changes in social acceptance over time. Wave 1 was launched before the introduction of the AVs; this initial survey established a baseline of public awareness, acceptance, and concerns regarding AVs^25^. Wave 2 was conducted nine months after the start of the shuttle service, the second wave aimed to capture the immediate impacts of the service on public perception, including any shifts in attitudes or concerns^26^. Wave 3, serving as the basis for the replication in Valais-Wallis, is described in the next subsection^27^. The response rates across the three waves were indicative of a high level of community engagement and interest in the topic. The first wave served as the baseline, with subsequent waves building on this initial engagement. Participants who completed the first survey were invited back for the subsequent waves, allowing for a longitudinal analysis of changes in individual perceptions over time. Data analysis focused on comparing responses across the three waves to identify trends and shifts in social acceptance. This approach facilitated a nuanced understanding of how direct exposure to the AVs influenced its acceptance more broadly. Such a structured approach to data collection and analysis provided a comprehensive overview of the public’s evolving acceptance regarding the AVs in Neuhausen am Rheinfall. By employing a longitudinal panel survey design, the study offered valuable insights into the dynamics of public opinion in the context of emerging transportation technologies, contributing to the broader discourse on the integration of AVs into public transport systems.

### The third survey wave in Schaffhausen and its replication in Valais: Comparing two Swiss cantons

The final survey, in wave 3, was carried out in Schaffhausen from August to October 2019, that is 18 months after the “Route 12” service began^27^. The approach for data collection offered self-administered questionnaires (online or paper) to accommodate participant preferences and ensure inclusiveness. This methodological choice was aimed at maximizing response rates and reducing potential biases associated with digital divide issues. Respondents were incentivized with a monetary reward to encourage participation across all three waves, enhancing the longitudinal study’s integrity through sustained engagement.

The third wave of the Schaffhausen survey served as the basis for a replication of the research in Valais. Invitations were sent to 4 000 random adults in three municipalities: Sion (the capital city of the canton), Sierre, and Martigny. The participation rate was 13.9% with 555 responses.

### Recruitment of survey respondents

In both cases of Schaffhausen and Valais, we employed a stratified random sampling approach. Half (50%) of the respondents were invited from the towns where the pilots were conducted, i.e., Neuhausen am Rheinfall (Schaffhausen) and Sion (Valais). The remaining 50% of respondents, divided equally among two strata (i.e., 25%), were invited from two adjacent towns to the one where the pilot was conducted. In the Canton of Schaffhausen, the towns were Stein am Rhein and Thayngen, and in the Canton of Valais, the towns were Sierre and Martigny. In all these cases, we requested a randomized list of respondents with postal addresses from the respective town halls and municipality offices of the aforementioned towns. We provided the municipalities with the necessary inclusion criteria to qualify a citizen as a respondent (e.g., 18 years and above, and registered residence). Additionally, we asked the municipalities to give us a list of potential respondents that included an equal number of male and female citizens. We received a randomized list of 2’000 potential respondents for the towns of Neuhausen am Rheinfall (Schaffhausen) and Sion (Valais). Additionally, the towns of Stein am Rhein and Thayngen (Schaffhausen), as well as Sierre and Martigny (Valais), provided us with randomized lists of 1’000 respondents each.

Next, each person on the randomized list was invited to participate in the survey via postal mail. The letter explained the purpose of the survey and introduced the research team and the institutes involved in the study. Participants were invited to take an online survey using a unique URL and password provided in the letter. Participants were also given the option to request a paper survey by indicating this preference on the URL provided in the letter. In such cases, paper surveys were mailed to the intended recipient, along with a prepaid envelope for returning the completed survey. Due to Switzerland’s multilingual landscape and the primary languages spoken in the two cantons of Schaffhausen and Valais, survey respondents could complete the survey in German, French, or English. However, the survey did not include the official Swiss languages of Romansch and Italian.

### Survey items and focus

The survey questions designed for the evaluation of the “Route 12” and “Smart Shuttle” pilot trials were developed to capture a broad range of factors influencing public perception, acceptance, and expectations of autonomous vehicles (AVs). Special attention was paid to aspects such as awareness of the service, perceived benefits and drawbacks, concerns about safety and privacy, and willingness to adopt or support AVs. The survey sought to assess residents’ perceptions not only of the technology itself, but also of its integration into public transport systems and its potential societal impacts. Specific items addressed the trial’s influence on public transport provision, the economy, traffic safety, environmental protection, and overall quality of life for residents.

The survey instrument (see files “Questionnaire Schaffhausen” and “Questionnaire Valais” in the Zenodo repository^30^) was designed to capture multiple dimensions of public perception. Items were either newly developed to reflect the specific Swiss pilot context or adapted from validated scales and prior studies, ensuring both contextual relevance and comparability with existing literature. General attitudes toward AVs and the pilot trials were assessed using items adapted from Kyriakidis, Happee & de Winter^31^ and Becker & Axhausen^32^. These items were intended to capture overall acceptance or rejection of AV technology and pilot projects, providing a baseline measure of public receptivity. Awareness of and direct experience with AV services were captured through newly developed items specific to the local context. These items reflected the documented influence of exposure on acceptance^25^, allowing analysis of how familiarity and direct interaction with the shuttles shaped perceptions.

User experience and perceived service quality were evaluated through items adapted from the Swiss Mikrozensus Mobilität und Verkehr 2015^33^. These items assessed aspects of ride quality, reliability, and satisfaction, providing insight into experiential factors that can affect adoption and acceptance. Safety, ease of use, and trust in technology were assessed using items based on Kyriakidis *et al*.^31^ and the R-1 risk perception scale^34^, reflecting core dimensions that have been shown to strongly influence willingness to adopt AVs. By including these measures, the survey could capture not only cognitive attitudes toward AVs, but also affective responses related to perceived risk and confidence in technology.

Broader societal impacts were explored through items newly formulated for the survey but conceptually linked to established frameworks such as the Technology Acceptance Model (TAM) and the Theory of Planned Behavior (TPB)^35,36^. These items captured respondents’ perceptions of the externalities of AV deployment, including potential effects on urban mobility, environmental sustainability, and community well-being. Privacy, regulation, and governance concerns were measured using items adapted from Kyriakidis *et al*.^31^ and the broader literature on technology governance, providing insight into potential institutional and regulatory barriers that could influence public acceptance.

Individual differences in technology attitudes and personality traits were also considered. Items adapted from the Technology Commitment Scale^37^ and the BFI-10^38^ captured technology commitment, openness to new experiences, and perceptions of technology-related anxiety. These measures allow for the analysis of heterogeneity in acceptance and adoption intentions, linking behavioral intention with underlying personality traits. Socio-demographic and mobility behavior variables were collected using standardized items from the BFS Mikrozensus 2015^33^ and a previously conducted mobility study in Switzerland^39^. This enabled stratified analyses along age, gender, education, income, region, and political orientation, supporting both the generalizability of findings and meaningful comparisons across studies.

The surveys in both cantons (the third wave study in Schaffhausen and the survey in Valais) used identical sets of core questions, with only minor modifications to account for local specifics of the pilot trials. By including items either newly developed for the Swiss context or adapted from validated sources, the dataset provides a reliable measure of public perception and acceptance while remaining comparable with prior studies. Although the study was not designed to validate models such as TAM or TPB, the conceptual alignment of survey items with key theoretical dimensions, combined with reference to established instruments, strengthens the dataset’s validity and provides a robust foundation for interpretation and cross-study comparison.

Overall, the survey design allowed for a comprehensive assessment of residents’ perceptions of AVs, combining measures of awareness, attitudes, experience, perceived risks and benefits, societal impacts, personality traits, and socio-demographics. This integrated approach ensures that the study can support nuanced analyses of acceptance, identify potential barriers to adoption, and inform evidence-based policy and technology development decisions in the context of Swiss pilot trials.

### Data processing

We have combined the data from the two surveys described above in 2.2, i.e., the data corresponding to the third wave of the Schaffhausen survey and the one survey conducted in Valais. In combining the data from the two surveys, we first selected the questions that were common to both surveys, mainly because both surveys had several questions that were specific to the region or service provider (see files “*Questionnaire Comparison*”, “*Questionnaire Schaffhausen*” and “*Questionnaire Valais”* in the Zenodo repository^30^). Next, we homogenized the directionality of survey questions that collected citizen responses on a Likert scale by ensuring that the order of response anchors (e.g., *[Strongly Disagree … Strongly Agree], [Negative … Positive]*) was the same for all questions. This included aligning responses that used different anchors, such as *[very bad … very good], [rather negative … rather positive]*, so that higher values would consistently correspond to agreement or a positive appraisal of the questionnaire item. Each response to a Likert scale question in our survey was assigned an integer value in the range *[1… 5]*. Moreover, some questions gave respondents the option of answering *“I don’t know”*. For these questions, a negative integer value *(−99)* was assigned when respondents selected this option so that we could easily identify them or apply filters during the analysis. The way the survey was organized also allowed respondents to opt out of answering questions, and these cases are represented as missing values in the dataset.

Five survey questions, which corresponded to perceptions of the shuttle (e.g., comfort and spaciousness) and the service (e.g., reliability, usefulness, and time efficiency), were recoded from a *[Very Bad … Very Good]* scale to an integer scale ranging from 1 to 5. Moreover, thirteen survey questions related to the role of autonomous shuttles in urban mobility (e.g., inclusiveness for commuters with disabilities, the elderly, and children; affordability; environmental friendliness, etc.) were transformed from a *[negative … positive]* scale to an integer scale ranging from 1 to 5. In addition, five questions related to perceived safety and ease of use offered respondents the option to answer *“I don’t know”*. In cases where this response was provided, we assigned a value of *−99* to the corresponding survey item.

### Ethics Statement

The survey studies conducted in the canton of Schaffhausen received ethics approval from the ETH Zürich Research Ethics Board, approval number EK 2018-N-01. The ethics committee approved the study protocol, including participant recruitment procedures, survey instruments, and the collection, processing, and sharing of anonymized research data. All participants received written information about the purpose of the study, the voluntary nature of participation, and the handling of their data. Written informed consent was obtained from all participants prior to participation. Consent included agreement to the use of anonymized survey responses for scientific analysis and open data sharing via a public research repository.

For the Schaffhausen surveys, access to postal address data from municipal population registries was additionally approved by the cantonal data protection authority of the Canton of Schaffhausen. A formal data protection agreement was signed with the cantonal data protection officer of the Canton of Schaffhausen, specifying that address data were to be used exclusively for survey recruitment purposes and permanently deleted after completion of data collection.

The survey conducted in the canton of Valais used the same survey instrument and followed the same study protocol as the third wave of the Schaffhausen survey, with only minor contextual adaptations related to the local pilot trial. It was therefore conducted in accordance with the same ethical principles and data protection standards as approved for the Schaffhausen study. The study received approval from the Data Protection Officer of the Canton of Valais, based on the prior authorization granted by the Data Protection Officer of the Canton of Schaffhausen.

## Data Record

All data and research materials are available in the Zenodo repository^30^: 10.5281/zenodo.17701663

The Zenodo repository^30^ contains several files, including one file containing the survey data (as a.csv file) and supporting files which are described below:

- **Data File** (*SmartShuttle-Survey-Final.csv*): All survey data from Schaffhausen and Valais cantons of Switzerland (as described above) is uploaded in the Comma Separated Values (CSV) format. Each row corresponds to a unique survey respondent and each column corresponds to an item in the survey (e.g., respondent’s gender, age, educational level, as well as their perception of the driverless shuttle). The column with the header title of ‘ID’ contains the unique and anonymous identifier of each respondent. The following 13 columns correspond to the demographic variables of the survey participants, including their gender, age, employment status, household and care responsibilities, and the city of residence of the respondent. In addition, two columns correspond to the ‘Language’ (EN = English; DE = German; FR = French) of the survey and the ‘Canton’ (SCH = Schaffhausen; VS = Valais) where it was administered.
  The remaining columns correspond to the respondents’ perception of the shuttle and of the service. Among these items (variables), the name of the column indicates the type of value. For instance, if the column name ends with ‘Likert’, it corresponds to an ordinal Likert scale value which lies in the range *[1 … 5]* (as illustrated in the methods section). A column name ending in ‘Binary’ indicates an item gauging a ‘Yes/No’ response. Furthermore, a column name ending in ‘Categorical’ signifies a nominal variable which registers a single value in a pre-defined set of nominal values. The mapping of column names to the respective item in the survey can be found in the file named “*Questionnaire Comparison*” in the Zenodo repository^30^, which provides the reader with the relevant questionnaire items in English, and maps them to the variable names used throughout the text and in the published data.
- **Data Mapping Table** (“*Questionnaire Comparison*” in the repository^30^): All complementary details about each dataset column (survey item and demographic variable) are contained in this spreadsheet under the sheet entitled ‘Appendix’. It maps each dataset column to the corresponding item in the Schaffhausen and Valais survey, including the type of data (Categorical, Likert, etc.). Each column name from the dataset (.csv file illustrated above) can be found under the ‘Dataset Variable’ column. Corresponding to each dataset variable is the unique English survey item identifier (Questionnaire_variable), the question/item text (in English, German, and French), and the question/item number (Q#) in both the Schaffhausen and Valais surveys. It is worth noting that there is an N/A value corresponding to the demographic questions in the survey. This is because we did not assign a unique identifier for these items and the file also contains supporting information.
- **Schaffhausen Questionnaire** (“*Questionnaire Schaffhause*n” in the repository^30^) This Microsoft Word (.docx) file contains the editable version of the German language survey which was administered in the third wave in the Canton of Schaffhausen, as illustrated above in the Background & Summary Section.
- **Valais Questionnaire** (“*Questionnaire Valais*” in the repository^30^): This Microsoft Word (.docx) file contains the editable version of the French language survey which was administered in the Canton of Valais, as illustrated in the Background & Summary Section.
- **Example Analysis Script** (*SmartShuttle-Script-Analysis.R*): This script, written for the R language (a free, open-source programming language used for statistical computing, data visualization, and data analysis), contains code snippets and examples enabling readers to load the aforementioned data file (.csv) in their workspace, describe the different dataset variables, illustrate their type, and also display descriptive statistics about the survey responses. In addition, the script also contains code snippets to conduct data wrangling and use basic statistical procedures to make inferences about the variables and the correlations between them.

## Data Overview

The final dataset contained 1361 responses to over 84 survey items, including socio-demographic questions. It should be noted that since many of these questions were not mandatory and some surveys were conducted on paper, there are missing values in the dataset. Furthermore, our data included 849 (62.4%) responses from the Schaffhausen survey and 512 (37.6%) responses from the Valais survey. There were also 848 (62.3%) responses to the German survey, 495 (36.4%) responses to the French survey, and 18 (1.3%) responses to the English survey in both cantons. Educational attainment varied, with 489 participants (36%) having completed vocational apprenticeships or schooling, and 269 participants (20%) holding university degrees. Additional educational backgrounds included higher technical or vocational education (223 participants, 16%) and universities of applied sciences or teacher education institutions (159 participants, 12%). Smaller groups reported high-school diplomas or vocational baccalaureates (93 participants, 7%) and mandatory schooling (62 participants, 5%). Participants’ ages range from 18 to 95 years, with a mean age of 53.5 years. Gender distribution indicates that 761 participants identified as male and 597 as female. Employment data reveals a variety of categories, with the most represented being employed in the private sector, with 495 participants.

The survey contained Likert scale items (or questions) that captured respondents’ perceptions and experiences of AVs and the service, i.e., its use in public mobility. The survey questions mainly asked respondents about their perceptions of the reliability, acceptance, usefulness, safety and cost-effectiveness of using autonomous shuttles as public transport. Respondents were also asked about their experience of the shuttle itself, i.e. their perception of its spaciousness, its reliability in unexpected and mixed traffic situations and bad weather, the safety of schoolchildren and pedestrians, and whether it was designed with people with disabilities in mind. The survey also probed respondents’ awareness of the basic operation of the AVs and their knowledge of any government regulations relating to them, in particular the need to establish regulations on data transfer, insurance and liability relating to AVs in Switzerland. Finally, respondents were asked about their views on future projections of AVs, including sustainability and its impact on socio-economic aspects such as job losses and wider social acceptance of innovative projects. See files “Questionnaire Comparison”, “Questionnaire Schaffhausen” and “Questionnaire Valais” in the Zenodo repository^30^ for a detailed list of items included in our survey.

## Usage Notes

The survey data offers a snapshot of social acceptance in two regions of Switzerland regarding the use of AVs as a public transport alternative. A blog post presenting the insights of the survey discussed the mixed perceptions of autonomous vehicles in urban areas, highlighting both the enthusiasm for their potential benefits and the concerns about safety and data privacy^40^. It is worth noting that these examples of AVs operating on roads in mixed traffic were the first of their kind in the world. Consequently, the survey also corresponds to the initial perceptions of AVs and provides an ecologically valid benchmark to compare the evolution of social acceptance in other geographic contexts or similar experiments with AVs in public transport.

In addition to providing a comprehensive examination of the social acceptance of AVs across multiple dimensions, such as perceived safety, relevance, desirability, utility, accessibility, and effectiveness, the survey also offers researchers the opportunity to examine relationships between survey variables and differences across various demographic characteristics (geographic context, language, etc.). For example, in our survey, there was a significant difference in the social perception of the AVs between the two cantons in terms of its accessibility for passengers with disabilities (Kruskal-Wallis: ***χ***2*(1)* = *77.40, p <* *0.001*), with respondents from the canton of Valais reporting a higher perceived accessibility of the shuttles, despite the use of the same vehicle in both cantons. Similarly, respondents in the canton of Valais rated the benefits of these self-driving shuttles for children significantly higher than those in Schaffhausen (Kruskal-Wallis: ***χ***2*(1)* = *37.01, p < 0.001*).

The analysis of the three panel waves conducted as part of the Route 12 pilot experiment in Neuhausen am Rheinfall provides comprehensive insights into public perceptions of autonomous driving technology. More importantly, they also indicate how the dataset can be used for comparisons with other regions and pilot trial contexts. Results show that awareness of the trial increased significantly over time, from approximately 70% in the first wave to over 90% by the third, indicating the growing visibility of the project among the local population. The findings further indicate that while familiarity effects on public opinion were limited, overall acceptance of the self-driving bus trial remained high and stable throughout the study. Although initial concerns, such as system reliability and loss of control, were robust predictors of lower acceptance, these concerns did not significantly diminish over time with exposure to the trial^25–27^. Additionally, an analysis revealed that external events, such as the widely publicized fatal accident involving a self-driving vehicle in Arizona, had a significant short-term negative impact on social acceptance. This accident heightened concerns related to safety and reliability, illustrating the sensitivity of public opinion to such incidents. Despite this, the negative effects were short-lived, and acceptance levels rebounded, highlighting the resilience of overall support for AVs when implemented in controlled settings. At the same time, a local non-fatal accident revealed no effects on acceptance levels^41^. These findings underscore the importance of addressing public concerns proactively while managing the narrative around unexpected events to sustain trust and support for disruptive technologies^29^. A mode choice experiment revealed that technology acceptance was a significant predictor of the use of AVs, with users demonstrating a preference for the service under specific conditions, such as shorter travel times, lower costs, and less crowded buses. However, overall willingness to pay for the service remained relatively low compared to other modes of public transportation, highlighting the importance of improving the perceived value of such services^28^ (replication data for the published papers are available at 10.3929/ethz-b-000392008 and 10.3929/ethz-b-000391197).

## Technical Validation

As the dataset contains responses to a public survey completed either on paper or digitally, the key survey items gauging social acceptance of autonomous shuttles were not mandatory. This resulted in missing values for these items in the dataset. Moreover, when preparing the dataset that merged responses from both digital and paper-based versions, real-time validation rules were incorporated. These included range restrictions for numerical items, predefined categories for multiple-choice questions, and automated completeness checks. For paper-based surveys, these checks were conducted manually during data entry by two co-authors independently. Finally, missing values and invalid entries (occurring mostly in paper surveys) were identified and replaced with ‘NA’ values corresponding to each specific item and respondent.

Besides testing for completeness and reliability of the data as mentioned above, we did not conduct statistical tests to assess the internal consistency of the survey items or the criterion and construct validity of these items. This decision was made because the purpose of the research was to capture social perceptions of AVs in two distinct cantons in Switzerland, rather than to assess the underlying constructs that influence these perceptions.

## Data Availability

All data and research materials are available at Zenodo^30^: 10.5281/zenodo.17701663.

## Code availability

The analysis code is available at Zenodo^30^: 10.5281/zenodo.17701663.
